# Surgical correction of pulmonary artery aneurysm with extrinsic compression of the left main coronary artery: A case report

**Published:** 2020-01-18

**Authors:** Artur Henrique de Souza, Pedro Arthur Ferreira Borges, Victor Eduardo de Almeida e França, Leonardo Veloso do Amaral, Geraldo Paulino Santana Filho, Jader Bueno Amorim, Marvyn de Santana do Sacramento, Jefferson Petto, Giulliano Gardenghi

**Affiliations:** ^1^Department of Cardiovascular Surgery, Bahia, Brazil; ^2^Department of Clinical Cardiology, Bahia, Brazil; ^3^Department of Cardiovascular Surgery, Holy House of Mercy of Goiânia, Goiânia, Goias, Brazil; ^4^Department of Program in Physical Therapy, Bahia University Social Center, Bahia, Brazil; ^5^Department of Physical Therapy, Bahian School of Medicine and Public Health, Salvador, Bahia, Brazil; ^6^Department of Physical Therapy, Bahia Adventist College, Cachoeira, Bahia, Brazil; ^7^ Department of Scientific Coordination, ENCORE Hospital, Aparecida de Goiânia, Goiânia, Brazil

**Keywords:** aneurysmectomy, angiography, angioplasty, morbidity, stent, surgery

## Abstract

Pulmonary artery aneurysm (PAA) is a rare disease, with a poorly known natural history, complex diagnosis and may evolve with serious complications, such as compression of adjacent anatomical structures. In some cases, the presence of such complications is what determines the symptoms and is the initial manifestation of the disease. This paper aims to report the case of a patient with typical angina, submitted to cardiac catheterization, which showed, among other lesions, severe left main coronary lesion with characteristics that led to the suspicion of extrinsic compression, which was identified as caused by a PAA. The treatment chosen in this case was surgical, reported concurrently with a literature review that guided the medical team in their decision-making.

**Relevance for patients:**

Surgical correction of PAA may provide resolution of coronary symptoms in affected individuals.

## 1. Introduction

Pulmonary artery aneurysm (PAA) is a rare condition, first described in 1860, whose prevalence, based on a series of necropsies, is expected to be one in 13,696 individuals [1]. The natural history of the disease is poorly known, and despite its rarity, it may be associated with several causal factors, thus presenting different evolutions [2]. They often have an indolent course and may, in some situations, lead to relating complications, such as compression or rupture of adjacent structures, increasing the morbidity and mortality rate. In such situations, the approach chosen is often surgical.

It is proposed to present an example of this situation in the present case report, evaluated by the research ethics committee of the Goiânia Emergency Hospital, and linked to Plataforma Brasil and receiving its approval under number CAAE: 08 498819.8.0000.0033.

## 2. Case Report

A 67-year-old female patient looked for cardiology service complaining of precordial pain beginning 3 weeks ago, in tightness, intermittent and associated with respiratory distress on medium exertion. She had hypertension, dyslipidemia, overweight, and mild gastritis due to comorbidities and was taking rosuvastatin, acetylsalicylic acid, metoprolol, and lansoprazole. Her physical examination showed no other noteworthy changes. The electrocardiogram presented sinus rhythm, anterosuperior divisional block and diffusely inverted T-wave. She brought stress echocardiography without segmental changes.

**Figure 1:**
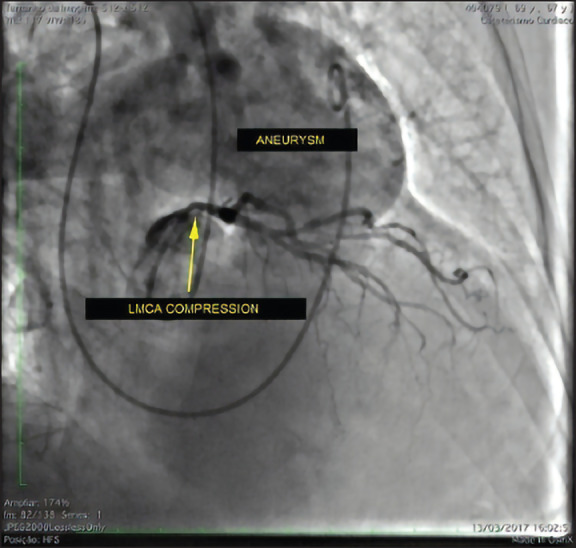
Angiography showing the pulmonary artery trunk, compatible with aneurysm, and suspected coronary main compression.

Due to the high risk of the patient, she underwent coronary angiography, and a significant obstructive lesion in the left main coronary artery (LMCA) was identified, without calcification and with a “pencil point” appearance and moderate lesions in the anterior descending (AD) and right coronary artery, no flow changes. During the examination, contrast-injected right chamber catheterization was performed, with no suspicion of alteration in the right atrium and ventricle, and pulmonary artery trunk enlargement, compatible with aneurysm, and suspected coronary trunk compression (Figure 1). The right chamber pressures were verified, with pulmonary trunk pressure of 35 mmHg.

**Figure 2:**
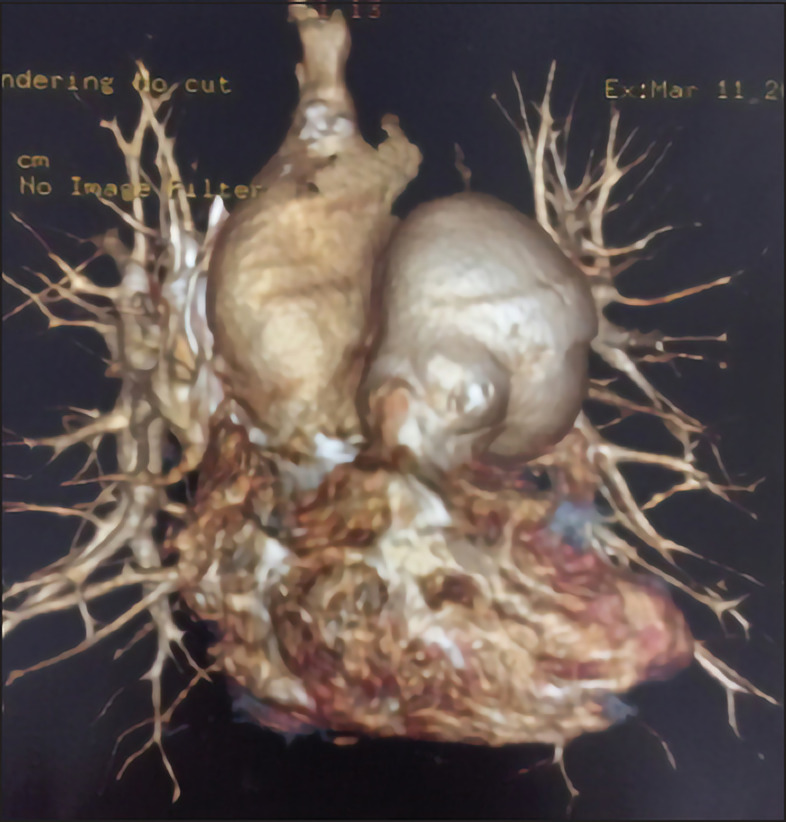
Coronary angiotomography showing pulmonary main artery aneurysm measuring 50 mm, identifying extrinsic compression of the left main coronary artery.

The patient underwent coronary angiography, identifying a pulmonary artery trunk aneurysm measuring 50 mm and finding extrinsic compression of the LMCA (Figure 2).

**Figure 3:**
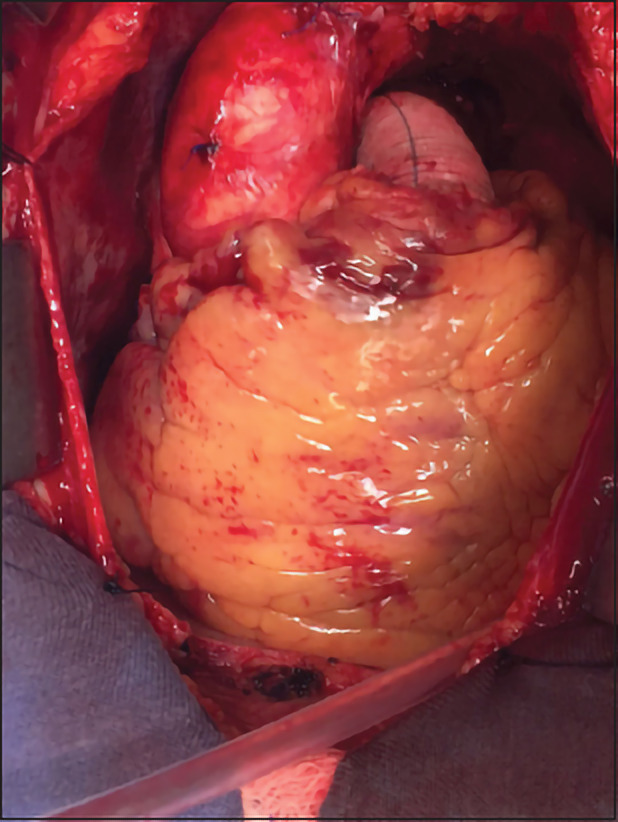
Complete resection of the pulmonary main artery up to the confluence of the right and left pulmonary branches with Dacron tube implantation.

In the present case, surgical correction of the pulmonary artery trunk aneurysm was indicated. The procedure was performed by median sternotomy and after pericardiotomy and full heparinization; cardiopulmonary bypass was started with the venous return through the right atrium and arterial return in the distal descending aorta under normothermia. After aortic clamping, a cardioplegic solution (modified St. Thomas) was performed and a complete resection of the pulmonary artery trunk was performed until the confluence of the right and left pulmonary branches with Dacron tube number 28 (Figure 3). After the removal of the aortic forceps and return of the heartbeat, hemostasis was reviewed and subsequently closed by planes. The post-operative evolution was satisfactory, and the patient was discharged on the 5^th^ post-operative day, in good general condition, with an improvement of symptoms related to the compression of the LMCA.

In the present case, surgical correction was chosen when establishing the anatomical diagnosis, finding a pulmonary artery base pressure measurement of 35 mmHg and the size of the aneurysm since its growth determined extrinsic compression of the LMCA to an extent sufficient to determine angina symptoms and ischemic alteration in functional cardiac test. Coronary angiography revealed a lesion in the middle third of AD coronary artery, without significant obstruction and without indication of approach at the time.

In the outpatient follow-up, symptoms recurred, and the patient underwent a new catheterization 16 months after the surgery when an increase in the degree of obstruction in AD was identified, treated with angioplasty and implantation of a pharmacological stent, and angiographically confirmed LMCA patency when compared to pre-operative control.

## 3. Discussion

Although it is a rare disease, PAA has different causal factors. High-pressure aneurysms may be idiopathic, associated with congenital heart defects – the most commonly reported are persistent ductus arteriosus and ventricular or atrial septation defects – or presented as a complication of an adjacent cause of pulmonary hypertension, most often due to thromboembolism [2,3].

Congenital heart abnormalities may also cause low-pressure PAA due to high pulmonary flow. Other causes of low-pressure aneurysm include post-stenotic or idiopathic dilatation and vessel wall changes in diseases such as Marfan, Ehlers-Danlos syndromes, and Beçet’s disease [3,4]. Pseudoaneurysms may occur due to these same vessel wall abnormalities or due to acquired causes such as adjacent neoplasms and fragility secondary to intravascular procedures such as Swan-Ganz catheter implantation [2,4].

PAA presents a varied spectrum of clinical manifestations, influenced among other factors by aneurysm pressure, size, growth velocity, and adjacent disease [2]. In situations with low pressure and slow growth, the patient may remain asymptomatic for years to decades [5]. In symptomatic cases, it may present with dyspnea, chest pain, hoarseness, palpitations, and syncope [3]. In some situations, clinical manifestations are secondary to the clinical complications of pulmonary aneurysm, including cyanosis, cough, progressive dyspnea, and bronchiectasis in patients with bronchial compression [3]; hemoptysis, rapid onset of circulatory shock in patients with aneurysm rupture [2]; and typical chest pain in individuals with extrinsic coronary compression [6].

In the presence of symptoms compatible with PAA, the suspicion may be increased with the presence of the right ventricular overload on electrocardiogram and hilar enlargement on chest radiography [2]. However, these changes may be absent [7]. Angiography is an important examination defining anatomy and pressure measurement, with caveats for its invasive characteristic and technical difficulty in performing [2,7]. Angiotomography, in turn, presents high accuracy and adequate definition of the anatomy [2]. The diagnosis of extrinsic compression of the LMCA can be a challenge, due to the dynamic characteristic of compression, and therefore, depends on an adequate diagnosis of PAA [6].

The lack of guidelines guiding the best time for surgical approach has been a challenge in cases of PAA; however, literature reviews indicate that pulmonary artery pressure and diameter measurements, as well as its annual growth rate, have been important in determining the risk of aneurysm rupture and thus establish the indication for surgical correction since this complication presents high mortality. Limit values found for surgical planning are 50 mmHg for pulmonary artery pressure, 75 mm diameter, and annual growth rate greater than 2 mm [3].

In individuals with PAA with extrinsic coronary artery compression, determinants of higher risk for ischemia were identified, such as the ratio between the pulmonary artery and aorta diameters greater than 2 and the angle between the LMCA and left sinus of Valsalva <30^th^ [6,8]. The patient’s ischemic assessment by functional testing may be important for the decision of invasive approach [6].

The invasive approach may differ depending on the cause of the pulmonary artery dilation and the degree of cardiac involvement. In clinical situations with low aneurysmal pressure, aneurysmorrhaphy may be the therapy of choice, with a low rate of intraoperative complications, with the exception that parietal stress may remain [5]. In patients with adequate clinical and anatomical conditions, arterioplasty with or without pulmonary valve replacement (depending on indication) is an advantageous method because it treats both aneurysm and extrinsic compression [9]. Synthetic graft aneurysmectomy, usually Dacron’s, associated with pulmonary valve replacement is the procedure of choice in cases with a higher risk of complications, such as pulmonary hypertension [2]. Myocardial revascularization may be performed in these situations, and in some reported cases, stent implantation was the treatment method of choice in patients with severe congenital disease associated with high surgical risk [8,10].

## 4. Conclusion

PAA is a low prevalence disease that can present as differential diagnosis of coronary obstruction and should be hypothesized in the suspicion of extrinsic compression of the coronary arteries. The proper diagnosis of this clinical condition should be combined with a thorough anatomical evaluation as well as pressure-1 and prognostic evaluation so that the need for a surgical approach is determined.
